# Echinobase: an expanding resource for echinoderm genomic information

**DOI:** 10.1093/database/bax074

**Published:** 2017-09-12

**Authors:** Parul Kudtarkar, R. Andrew Cameron

**Affiliations:** 1 Division of Biology and Biological Engineering, California Institute of Technology, Pasadena, CA, USA

## Abstract

Echinobase, a web accessible information system of diverse genomics and biological data for the echinoderm clade, grew out of SpBase, the first echinoderm genome project for sea urchin, *Strongylocentrotus purpuratus*. Sea urchins and their relatives are utilitarian research models in fields ranging from marine biology to developmental biology and gene regulatory systems. Echinobase is a user-friendly web interface that links an array of biological data that would otherwise have been tedious and frustrating for researchers to extract and organize. The system hosts a powerful gene search engine, genomics browser and other bioinformatics tools to investigate genomics and high throughput data. The Echinobase information system now serves genomic information for eight echinoderm species: *S. purpuratus*, *Strongylocentrotus fransciscanus, Allocentrotus fragilis*, *Lytechinus variegatus*, *Patiria miniata*, *Parastichopus parvimensis* and *Ophiothrix spiculata*, *Eucidaris tribuloides*. Herein lies a description of the web information system, genomics data types and content hosted by Echinobase.org. The goal of Echinobase is to connect genomic information to various experimental data and accelerate the research in field of molecular biology, developmental process, gene regulatory networks and more recently engineering biological systems0.

**Database URL:** http://www.echinobase.org

## Introduction

The first echinoderm genome to be sequenced and assembled was that of the purple sea urchin, *Strongylocentrotus purpuratus* (1). The draft assembly was completed using a whole genome shotgun strategy at the Baylor School of Medicine, Human Genome Sequencing Center (BCM-HGSC). This assembly was subsequently used to define a set of protein coding genes, which was then annotated by the research community through a web portal at the BCM-HGSC (2). These sequences and annotations became the first version of the sea urchin genome database, SpBase (3). As the annotation effort proceeded, sequence analysis showed the assembly to contain about 15% redundant sequences. This was undoubtedly due to the high level of genomic polymorphism in this species. Hence, a bacterial artificial chromosome strategy was used to produce large sequences of a single haplotype. Subsequently, additional sequencing platforms contributed more genome coverage and improved assemblies (4). At each increment of assembly improvement, the original gene predictions were mapped to the new assembly thus preserving the results of the large annotation effort.

The rational for sequencing projects stems from the general utility of certain echinoderm species, especially sea urchins, as research models in cell and developmental biology, with a history that extends back to the mid-1800s. This rich information base served the expansion of this system into the modern era of molecular biology. In the modern era, work with the purple sea urchin has focused on molecular mechanisms of development including how the genome directs the development of form. The most complete description of the gene regulatory network that drives the developmental processes of specification and differentiation is that of the purple sea urchin embryo. The underpinnings for the genome sequencing project began to be accumulated in earnest in 1998 (5).

Other echinoderm genomes are more recently being sequenced using next-generation methods in much shorter intervals of time. The genome information from additional species calls for a broader portal for genome information than that which SpBase could accommodate. A new web information system called Echinobase, http://echinobase.org has been constructed to fulfill this need. It is the subject of this report. The first version of Echinobase was made public in December 2013. Here we describe the genomes and species that contribute to this echinoderm genomic information base. We will discuss the extent of annotations that accompany the various genome assemblies and the other sorts of information that are associates with genomes and gene sets. We examine here the content and structure of the information system.

## Species

Extant echinoderm groups include crinoids, or feather stars and the four eleutherozaon classes: sea urchins (Echinoidea); sea stars (Asteroidea); sea cucumbers (Holothuroidea) and brittle stars (Ophiuroidea). Relaxed molecular clock estimates place the divergence of the four eleutherozoan classes in a brief period of about 5 million years starting about 500 million years ago (Ma) (6). Only a few echinoderm groups survived the End-Permian mass extinction and one of the two surviving echinoid lineages gave rise to the modern cidaroids and the other to the euechinoids (7). All of the known post-Permian echinoderms were small animals inhabiting shallow, well oxygenated seas (8).

The relationship between the ancient eleutherozoan classes is central to understanding the morphological evolution of this phylum and hence the value of its genome sequences. If the Ophiuroidea (brittle stars) are the sister group to the Echinozoa (sea urchins + sea cucumbers) (6), then the morphologically similar echinoid and ophiuroid larval forms are homologous not convergent as other phylogenies would suggest. Indeed, the most recently two analyses of echinoderm transcriptomes both favor the asterozoan topology where the sea urchins are most closely related to the sea cucumbers and the sea stars (Asteroidea) are most closely related to the brittle stars (7, 9).

In addition to its utility as a research model the purple sea urchin and echinoderms in general sit at particularly advantageous phylogenetic position for useful comparisons to other members of the deuterostome clade such as higher vertebrates. Together with the hemichordates, the echinoderms constitute a group designated Ambulacraria, the basal sister group to the chordates. Echinoderms have a well-populated fossil record stretching back to the Cambrian period and offer insights into the deuterostome origins and diversification. The Echinobase information system serves genomic information for eight echinoderm species.

## Datasets

### Genomes

In 2006, the draft genome assembly for an echinoderm species (*S. purpuratus*) was published (1). Four additional rounds of sequencing have been obtained for this species and now it is the most mature with 32 009 scaffolds and 50% of the sequence contained in scaffolds >431 Kb (Table 1). The most closely related species, *Strongylocentrotus fransciscanus* and *Allocentrotus fragilis*, were only sequenced to a depth of 2× genome coverage and are used primarily for comparison to *S. purpuratus*. There is insufficient sequence coverage to assemble into contigs. These sequences are provided as tracks on the *S. purpuratus* genome browser. The individual sequences can be recovered there. Next, two additional genome sequence assemblies were added: the variegated sea urchin from the East Coast of the US, *Lytechinus variegatus*, and the sea star from the Pacific coast of the USA, *Patiria miniata*. These species are actively used as research models in cell and developmental biology. The genome assembly of *L. variegatus* has been through two rounds of sequencing (Illumina and PacBio) arriving at a scaffold N50 of 46Kb. The *P. miniata* assembly remains as a first version with about 60 000 scaffolds and a scaffold N50 of 53Kb. It was assembled from 70× coverage of reads from the Illumina platform. Most recently assemblies for the sea cucumber, *Parastichopus parvimensis*, sea urchin, *Eucidaris tribuloides* and the brittle star, *Ophiothrix spiculata*, have been added. The differences in completeness for these assemblies results from varying amounts of sequencing, different sequencing platforms employed and the quality of the genomic DNA used (4). For example, *P. parvamensis* has about twice the scaffold N50 compared to *E. tribuloides* and the sequence coverage is 339x and 50.6× respectively.

**Table 1.** Genome assembly statistics for the six species included in Echinobas

Species	Spurp4.2	Lvar2.2	Etri1.0	Pmin1.0	Ppar1.0	Ospi1.0
Total sequence length	990 Mb	1061 Mb	2187 M	811 Mb	873 Mb	2764 Mb
Number of scaffolds	31 897	322 794	637 071	60 183	21 559	75 696
Scaffold N50	419 550	46 348	39 192	52 614	89 133	72 780
Number of contigs	146 295	452 418	1 006 568	179 756	150 862	644 798
Contig N50	16 785	9657	6630	9466	9587	6474

The unassembled low coverage genome sequence datasets for Sf and Af are not included. Abbreviation: Spurp, *S. purpuratus*; Lvar, *L. variegatus*; Etri, *E. tribuloides*; Pmin, *P. miniata*; Ppar, *P. parvamensis*; Opsi, *O. spiculata.* N50 refers to length such that scaffolds of this length or longer include half the bases of the assembly.

### Transcriptomes

At Echinobase are posted gene models assembled from deeply sequenced cDNA pools (RNA-Seq) made at various life stages of echinoderm species from the eleutherozoan classes. These gene models are accessible through a search form for the respective genome which returns a gene information page. Depending on the species, different methods were used to assemble the short reads. The completeness of the gene sets thereby created varied depending on the number of life stages sampled and the state of the genome used for assembly of the gene models.

The most comprehensive transcriptome is that of *S. purpuratus* which is based on 10 different embryonic stages, six feeding larval and metamorphosed juvenile stages, and six adult tissues (10). The set of 21 000 transcripts thus derived constitute a systematic upgrade of the gene model predictions throughout the genome. The transcript based gene model data were used to define average structural parameters for *S. purpuratus* protein coding genes. In addition, a custom sea urchin gene function ontology was constructed, and about 7000 different annotated transcripts were assigned to 24 functional classes (10). Strong correlations became evident between given functional ontology classes and structural properties, including gene size, exon number, and exon and intron size. The transcriptome sequence analysis and annotation has been added to the gene information segment of the web information system.

Predicted transcript datasets for the other echinoderm species for which we have genome assemblies are also posted at Echinobase. The Echinobase staff has generated draft gene model sets for these assemblies using Maker2, a genome annotation pipeline that functions well when training data is limited (11). Briefly, gene models are predicted from the repeat masked genome assembly. The gene model engine is trained using species-specific datasets that include transcriptome, expressed sequence tags (EST) and protein as well as evidence from HMM-based *ab initio* gene predictors such as SNAP gene (12) and AUGUSTUS (13). A repeat library used for genome masking was generated in house using RepeatModeler pipeline (Ernst et.al unpub.). The transcriptome evidence included RNAseq data assembled from 2.65 billion, 38 million and 171 million reads available for *L. variegatus, P. miniata*, and *P. parvimensis*, respectively.

These data are only a subset of the datasets for echinoderms that are listed at Genbank. As of this writing, there are 645 echinoderm entries in GEO Datasets and 1213 SRA experiments for echinoderms. The strategies used to derive these data include RNA-Seq, ChIP-Seq, ATAC-Seq and others. We provide a link to the data in Genbank for both repositories.

#### Annotation

The curated annotations for the GLEAN gene models constructed at BCM-HGC for *S. purpuratus* cover a total 29 886 gene models. This information was moved to Echinobase en mass. The information includes data on gene name, genomic location, pertinent literature, ontological annotations and expression information. We continue to improve the established gene annotations using the browser-based Web Apollo tool (14). The availability of the set of transcriptome-derived models based on the current assembly (10) has been crucial to this effort.

From the MAKER2 predicted gene models for other species were computationally produced annotations through best BLAST (Basic Local Alignment Search Tool) (15) hit against *S. purpuratus* transcriptome-derived models. These annotations are further validated by best Genbank hit and IPR scan to confirm the most accurate electronic annotation. Table 2 summarizes the number of annotated gene models for *S. purpuratus*, *L. variegatus, P. miniata* and *P. parvimensis* along with pipelines used for annotating these gene models.

**Table 2.** Number of annotated genes and annotation pipeline used for four species included in Echinobase

Species	Number of annotated genes	Prediction Type
Sp3.1	29948	GLEAN/manually annotated
Lv2.2	22105	Evidence based de novo prediction
Pm1.0	29697	Evidence based de novo prediction
Pp1.0	17379	Evidence based de novo prediction

Abbreviation: Sp, *Strongylocentrotus purpuratus*; Lv, *L. variegatus*; Pm, *P. miniata*; Pp, *P. parvamensis.* Sp uses GLEAN pipeline for gene annotation and Lv, Pm and Pp are annotated using MAKER2 pipeline.

## Website structure

**Figure 1.**
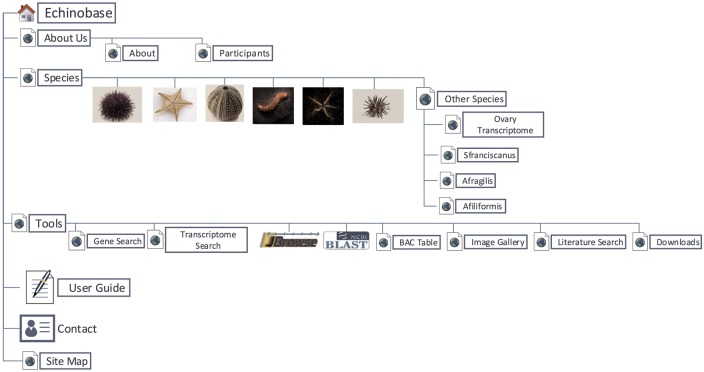
Organizational structure of Echinobase webpages. The primary menu items include species page, genomic and bioinformatics tools and user guide.

**Figure 2.**
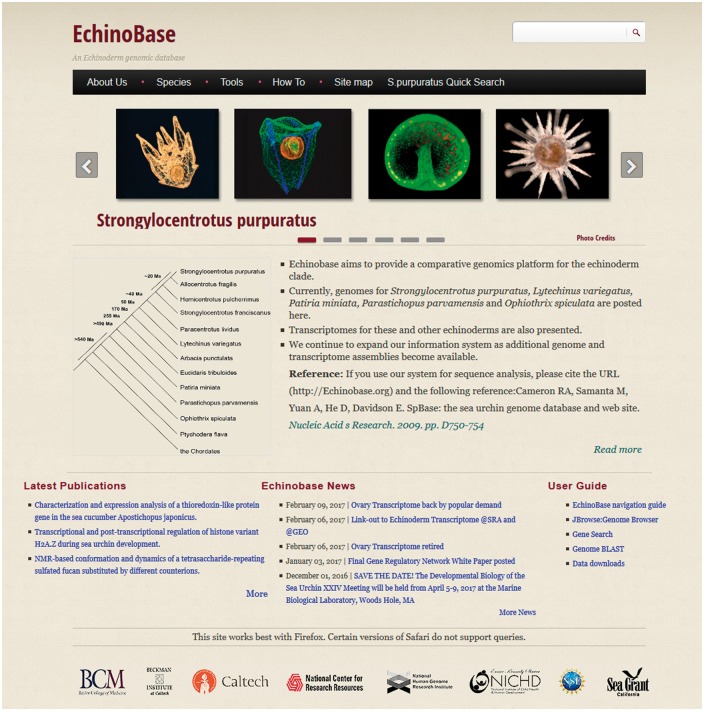
Echinobase landing page. A screen capture of the landing page from which drop-down menus lead to the various species and information groups.

**Figure 3.**
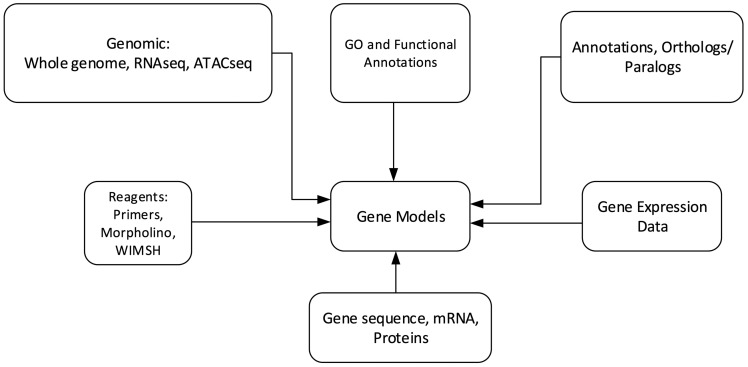
Schematic diagram of genomics and biological content stored in Echinobase. The relationships among the various data types are nominally arranged around the gene models.

Echinobase hosts a variety of information for the eight echinoderm species based first from genome sequencing. Figure 1 illustrates the organizational structure of Echinobase. The information posted includes the genome assemblies, predicted genes, transcriptome data and any known expression information. The organized information can be accessed through gene name or identifier search pages, the genome browser and a BLAST search engine (Figure 2). The genomics and experimental data-types stored in Echinobase are illustrated in Figure 3. Various data-types stored in Echinobase along with the tools and web information system that render the genomic content are explained in more detail in sections to follow.

### Gene and transcriptome search pages

The data-types illustrated in Figure 3 can be accessed from a gene name on the Gene Search Page. This search engine handles the dynamically generated annotation pages for 99 129 genes. A search query executes 41 database queries to populate the gene annotation page. Currently Echinobase has separate gene search utilities for *S. purpuratus*, *L. variegatus*, *E. tribuloides, P. miniata*, *P. parvimensis, O. spiculata* and an ‘Other Species’ category. Genes can be searched by Scaffold number, GLEAN Id, Gene Name, WHL Id (transcriptome Id), synonym and PubMed Id. The search engine flexibly handles the standard alpha-numeric wild card symbols. The Postgres pattern matching strategy returns all relevant hits for truncated queries. The hits from a search term are returned as a table that can be ordered by SPU ID, common name or gene name. The SPU ID of each hit links to a gene information page. Since *S. purpuratus* is more widely used for molecular biology research compared to other echinoderm species, its gene information system is much more extensive. Included here are data from a variety of high throughput experiments such as RNA-seq transcriptome measurements (10), whole embryo temporal expression from qPCR (16, 17) or from the Nanostring nCounter using a codeset of 172 regulatory genes. In addition, we link to separately maintained web accessible echinoderm information such as the gene expression site at the National Institute of Dental and Cranofacial Research (18).

**Figure 4.**
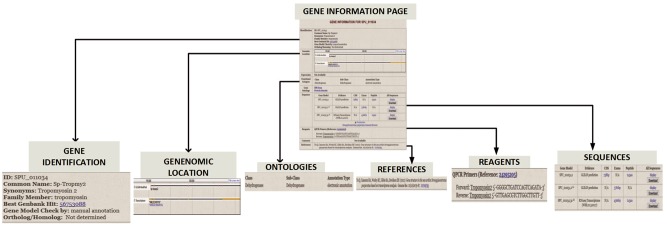
Gene Information page for *S. purpuratus* Tropomyosin gene. For each curated gene, the Gene Information Page displays a. gene identifiers such as synonym, family member and ortholog/homolog b. Genomic location on genome browser c. Ontology classification that includes functional category and GO terms d. gene sequence e. manually curated reagents and f. Pubmed references to the gene.

The Gene Information Pages serve as hubs to explore the information about a particular gene. The textual information displayed on the page are names, synonyms, various identifier symbols, Best Genbank Hit, Ortholog/Homolog information and GO classification. Links lead to (i) a page of gene expression information including links to high-throughput results, (ii) the results of protein motifs assignments to this gene (InterProScan), (iii) a genome browser snapshot that indicates genomic location, (iv) sequences, (v) reagent information and (vi) references by PubMed number. Figure 4 illustrates the gene information page for *S. purpuratus* aristaless-like homeobox 4-like gene.

Gene Ontology and InterProScan annotations are routinely updated to include latest releases for protein signature and ontology search database. Protein domain assignment come from superfamily (using the HMM library for assignment).

For each gene model coding, exon and peptide sequences can be viewed and downloaded as a fasta file. We include versions of gene model sequences that include: (i) GLEAN predicted genes (cds and peptides) (ii) GLEAN predicted genes with 3′UTRs (exons) and (iii) Transcriptome predicted genes (exons and peptides).

- Gene specific reagents and references are manually curated at Echinobase and updated at regular intervals. Reagent information includes QPCR primers, translational block morpholino-substituted antisense oligonucleotides, WMISH and RT-PCR primers along with a journal citation link. A subset of the original GLEAN [https://sourceforge.net/projects/glean-gene/] prediction models generated against the Spur0.5 assembly has been structurally re-annotated on the Spur3.1, the changes have been extensively logged into the comments section which typically indicates if a gene has a duplicate, isomer, overlapped ribosomal RNA or if gene model is derived directly from the RNAseq transcriptome. Gene references for published and publicly available Echinoderm research papers are regularly collected for updating the relevant literature repository.

### Genome browser

**Figure 5.**
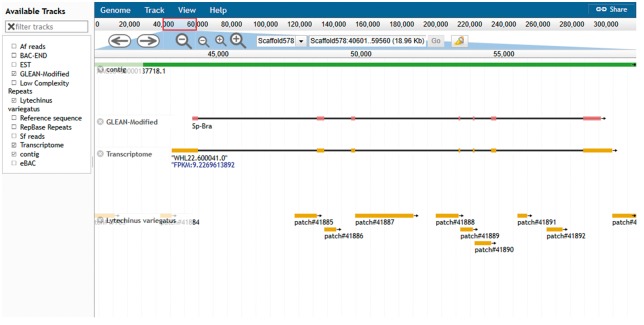
Echinobase genome browser example. A screen capture displaying the genomic region around the Sp brachyury gene. Individual tracks map genome assembly contigs, two versions of the gene model and conserved regions from the genome assembly of *L. variegatus* a related sea urchin to the reference genome sequence. Other tracks minimized in the screenshot are 1. BAC-END showing the location and coordinates of the sequenced ends of clones from the sperm genomic BAC library 2. eBACs track that displays the position of the deconvoluted individual BAC sequences enriched with WGS sequences 3. EST track showing the position of expressed sequence tags in the genome 4. the Repbase repeats track and low-complexity repeats track 5. mapped reads from phylogenetically closer genome of *A. fragilis* and *S. franciscanus.* The search field located at the top center of the browser can be used to search for gene symbol or scaffold coordinate such as illustrated in the figure.

The another resource is the genome browser, JBrowse (19). This browser supports easy navigation and allows the uploading of local files in many formats to complement the web system data. JBrowse is java-based software; it loads multiple tracks quickly and scales well for large regions. The genome browser for *S. purpuratus* displays the landscape of genomic features as linear tracks aligned to the reference sequence. The different features displayed include genome assembly scaffolds which serve as reference sequence, contigs, gene models from various sources, eBAC, BAC-END, RepBase and low complexity repeats. Mapped reads from closely related species *S. fransciscanus* and *A. fragilis* are available as is a set of tracks representing elements conserved between *S. purpuratus* (Figure 5). Other genomes such as *L. variegatus, P. miniataand P. parvimensis* have contigs, MAKER2 predicted gene models and genomic repetitive sequence tracks.

A survey of putative open chromatin regions in Sp embryonic stage nuclei determined by the ATAC-seq strategy are shown in a separate genome browser instance. The ATACseq Genome Browser for Sp displays possible open chromatin regions determined at 18, 24, 20, 39, 50, 60 and 70 hpf across three biological replicates (GSE95651). The ATAC-seq reads were computationally reduced using bowtie for read alignment across the genome and MAC2 for peak predictions. Tracks for gene models, contigs, and regions conserved with *L. variegatus*, and eBAC mappings are available in this browser. The linear arrangement of multiple tracks allows the user to manually infer features such as cis-regulatory modules from feature convergences. Linked track element data such as sequence and coordinates is available by right clicking on the feature track.

### BLAST search

We maintain an instance of the NCBI standalone BLAST server. Sequenced genomes such as those of *S. purpuratus, S. fransciscanus, A. fragilis, L. variegatus, P. miniata, P. parvimensis, O. spiculata, E. tribuloides* and Ovary transcriptome have NCBI BLAST powered search engine. BLAST search can be made against genome, contigs, gene models, proteins and RNAseq data (subjective to data availability).

### Textpresso for Echinoidea

Published and publicly available sea urchin research papers are regularly collected for updating the literature links and the reagent data in Echinobase. The literature data including title, abstracts, full length text etc. are also used for updating our literature database ‘Textpresso for sea urchin’. First, all the sea urchin research papers are fetched from PubMed. We use a scripts from the Textpresso (20) developing team and echinoderm specific keyword phrases to exhaustively search PubMed literature database for echinoderm related papers. An automated pipeline is used to retrieve positive hits followed by a manual step to retrieve those hits that are not reachable through PubMed directly. Finally, each paper is examined for relevance to Echinoderm research by the content of the abstract, and true sea urchin (Echinoidea) papers are selected. Literature resources are added to ‘Textpresso for sea urchins’ and filtered for useful reagents that are displayed on the gene information pages.

### Data downloads

The entire contents of Echinobase are available to the user through a Download portal accessible directly or through each Species page. Various legacy versions of sequence data are also maintained for download. The primary sequence data located here is archived at Genbank. As a policy we base our computational work and web information on the publicly archived sequences. In addition to sequence data, text dumps of our complete databases and the web information system code are reached through the Download portal. ‘How To’ pages explain the methods necessary to mount a local copy of Echinobase.

### Resources + materials and methods

As a gateway for the Sea Urchin Gene Library Resource, Echinobase also posts experimental methods and availability lists for BAC libraries and cDNA libraries generated for that resource. To aid the users of these physical resources the web site makes available methods such as library screening protocol, probe analysis, library preparation protocols and BAC library techniques. BAC library tool section lists >400 identified BACs and 98 recombineered BACs generated in-house at the resource. The identified BAC clones derive from all of the species in our collection and are listed by species name, gene name, spu id, transcriptome id, BAC name, reporter, NCBI link-out and length in base pairs (estimated during Sp0.5 sequencing project or pulse-field gel electrophoresis).

### Technology behind Echinobase

**Figure 6.**
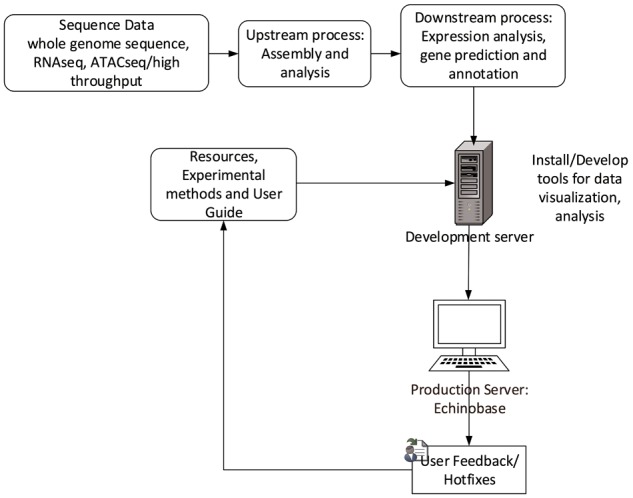
Echinobase data-flow. This diagram shows the bioinformatics workflow that produces the data displayed at Echinopbase. Computationally reduced data is hosted on development server that contains prereleased features for software testing. The stable, official content is hosted on publicly accessible production server www.echinobase.org.

Echinobase employs Drupal, an open source Content Management System (CMS). CMS simplifies website installation, construction and management for programmers and web content changes for nontechnical users. Its characteristics allows us to build structured content appropriate for a research model organism database (MOD). The Gene Search Tool is built outside Drupal and uses web-programing languages such as PHP, JavaScript and Cascading Style Sheet to generate dynamic and interactive graphical user interface. The database uses PostgreSQL database to store and organize gene information. The web development workflow follows staging genomics and experimental data to development server, followed by feature testing and debugging for corner cases and finally launching the feature to production server echinobase.org. Figure 6 indicates the Echinobase feature and data flow diagram. Git version control system is used to log revisions and maintain information integrity standards.

## Conclusions

Molecular research work in the genomic era requires a full-featured information system in support of the model organism in question. Gene and genome sequence data, gene expression information and sequence-based reagent descriptions are as indispensable to research today as are restriction enzymes and cloning vectors. Furthermore, these genomic data have reached a volume that is not manageable in an individual laboratory context. Next-generation sequencing also adds to this volume by blurring the line for the experimentalist between well-characterized research models and less well-known species. It is the task of genome databases to serve this kind of information to the experimental biologist in an efficient and easily accessible manner. This is the expressed goal of the Echinobase web information system.

The breadth and complexity of the data and organization of Echinobase falls in a middle position among MOD. This is a function of funding support, client interest, the nature of the organism and history. For example, research models such as the nematode *Caenorhabditis elegans* and the fruit flly, *Drosophila melanogaster* are very popular model systems and are amenable models to genetic manipulation. The data shared through their MODs reflects this additional information. Furthermore, many more groups study these models and therefore there is much a larger body of information on phenotypes and gene expression. In contrast, lower deuterostome models such as the hemichordate, *Saccoglossus kowalevski*, and the cephalochordate, *Branchiostoma floridae*, have a smaller research community and are not used for genetic studies. The smaller datasets for these animals is encompassed in a somewhat simpler database.

The utility of genomic and transcriptomic information is greatly enhanced by inter-species comparisons of many kinds. Today gene identification usually depends on sequence alignments with species both nearly and distantly related to the target. The software suite, BLAST, is probably the most highly used package in molecular biology and like other comparative sequence analysis methods it is based on an evolutionary model however tacit is the reference to that model. In addition to the algorithm of sequence comparison, some form of phylogeny is included in the evolutionary model. Much discussion still revolves around how species are related. Accurate phylogenies now often include both molecular and fossil evidence so that divergence distances can be calibrated with well characterized fossil chronologies. It is in this context that genome information systems and databases have grown from single species entities to sites covering a group of related organisms.
